# Trans-Atlantic collaboration: applying lessons learned from the US CF Foundation quality improvement initiative

**DOI:** 10.1186/s13023-017-0744-8

**Published:** 2018-02-08

**Authors:** Kathryn A. Sabadosa, Marjorie M. Godfrey, Bruce C. Marshall

**Affiliations:** 1 The Dartmouth Institute for Health Policy & Clinical Practice, Williamson Translational Research Building, One Medical Center Drive, Lebanon, NH 03756 USA; 2 Cystic Fibrosis Foundation, 4550 Montgomery Ave. Suite 1100 N, Bethesda, MD 20814 USA

**Keywords:** Cystic fibrosis, Clinical microsystem, Quality improvement program of care

## Abstract

**Background:**

Between 2002 and 2006 France launched a national cystic fibrois (CF) newborn screening program; organized a network of specialized CF care centers; and issued CF diagnostic and treatment standards. To continue to build on this success in 2007 the Cystic Fibrosis Center of Expertise for Rare Diseases (CF CERD) of Nantes-Roscoff in partnership with the French CF Society, the French CF Association (Vaincre la Mucoviscidose), and all CF center leaders from across the country agreed to pursue center-level improvement in medical outcomes for people with CF by adapting the U.S. Cystic Fibrosis Foundation’s (US CFF) national initiative, *Accelerating the Rate of Improvement in* CF *Care*. To launch the Program to Improve Results and Expertise in CF (le Programme d’Amélioration des Résultats et de l’Expertise en Mucoviscidose - PHARE-M), French leaders pursued mentorship and guidance from leaders at the US CFF, the Dartmouth Institute (TDI), and clinical care teams at CF centers across the U.S.

**Methods:**

The following activities enabled the Nantes-Roscoff CF CERD team members and a parent, involved with the French CF Association board and a quality engineer by training, to gain the leadership and quality improvement knowledge and skills necessary to implement the PHARE-M program: 1) regularly attending national meetings, tracking publications, and leveraging existing partnerships; 2) completing two sabbaticals to visit U.S. CF centers and enrolling in academic and professional training courses; and, 3) inviting US CFF and TDI leaders to France to meet key opinion leaders and frontline teams.

**Conclusions:**

The Nantes-Roscoff CF CERD team successfully adapted the US CFF’s initiative to accelerate improvement in CF care by establishing a partnership with U.S. leaders to communicate and exchange strategies and lessons learned; intentionally studying and adapting the Clinical Microsystems approach to quality improvement; and learning directly from the experience of frontline teams in the U.S. They continue to partner with U.S. leaders and are seeking to collaborate with European colleagues to continue to improve care for individuals with CF and their families across Europe.

## Background

Inter-professional healthcare teams at 124 centers, each accredited by the US CFF, deliver care to approximately 28,000 individuals with CF in the US. With patient consent, medical outcomes and data about the processes of care are captured and reported by way of the US CFF’s Patient Registry [1]. Variation in center-level pulmonary and nutrition medical outcomes, first reported in 1999, prompted the US CFF to launch a national improvement initiative, *Accelerating the Rate of Improvement in* CF *Care,* in 2002 [1, 2]. The aim of this ongoing initiative is to improve the quality and length of life for individuals with CF through the delivery of exemplary care at all centers. Goals such as individuals with CF and families (i) are full partners with their team of healthcare professionals, (ii) will have normal growth and nutrition, (iii) will receive appropriate therapies to maintain lung function and prevent exacerbations, (iv) are informed to prevent acquisition of respiratory pathogens, (v) are screened for complications to enable aggressive management, (vi) are supported in making decisions regarding transplantation and advance care, and (vii) will have access to treatments regardless of race, age and ability to pay, further define the initiative’s aim [3].

The initiative encompasses several key elements: a web-based patient registry to facilitate data capture and reporting; a quality improvement learning collaborative to teach leadership skills and improvement methods; a benchmarking initiative to identify and enable best practice; discipline-specific mentoring programs to connect healthcare professionals new to CF care with more experienced peers; public reporting of center-level data from the patient registry; publication of evidence-based clinical care practice guidelines; and, a framework for partnering with patients and families to improve care [1–7].

**Fig. 1:**
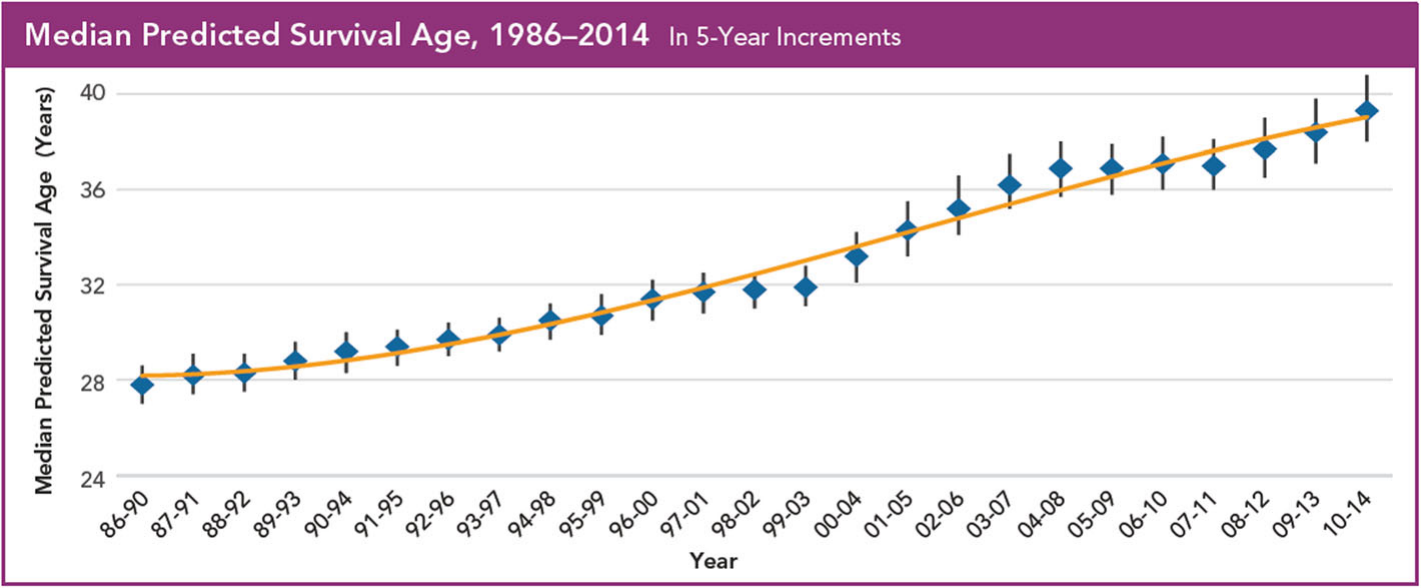
Median Predicted Survival Age as reported in the 2014 US CFF Patient Registry Report

**Fig. 2:**
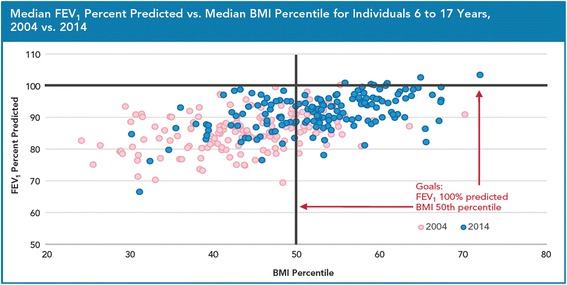
2004 vs. 2014 US CFF Accredited Center-Level Pulmonary and Nutrition Outcomes for Individuals with CF 6 to 17 Years of Age

**Fig. 3:**
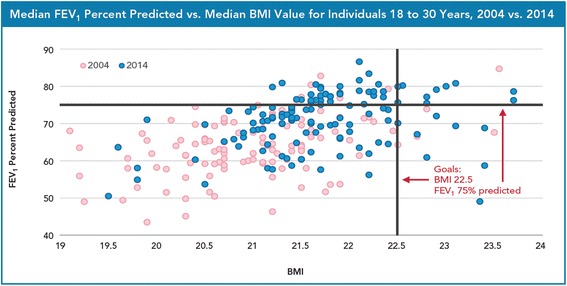
2004 vs. 2014 US CFF Accredited Center-Level Pulmonary and Nutrition Outcomes for Individuals with CF 18 to 30 Years of Age

Progress on each of the initiative elements coupled with the remarkable advances in basic science and therapeutic discovery led the US CFF to report a 10 year (31.3 years to 41.1 years) increased survival for individuals with CF between 2002 and 2012 [2]. The US CFF also reported improvement in median values for pulmonary and nutrition outcomes across all centers: median forced expiratory volume in 1 s (FEV_1_) percent predicted for individuals with CF aged 6 to 17 years in 2002 was 88.3 and increased to 94.3 in 2012; and body mass index (BMI) percentile for individuals with CF aged 6 to 17 years was 40.8 in 2002 and increased to 51.3 in 2012 [2]. Survival and center level results continue to improve as seen in the 2014 US CFF Center Directors Report (see Figs. 1, 2 and 3).

Over the course of executing the initiative, the US CFF regularly reported updates through national meetings and publications (www.cff.org) and routinely invited community members to participate via a number of opportunities ranging from accessing on-line material in the form of the CF Action Guide (http://clinicalmicrosystem.org/knowledge-center/workbooks/) to formal invitations to join a collaborative, serve on a committee, or enroll in a specific program.

In 2002 France launched a national CF newborn screening program which solidified the formation of a network of specialized CF care centers. There are 45 CF centers, geographically distributed by province and staffed by multidisciplinary clinicians (http://www.vaincrelamuco.org/face-la-mucoviscidose/les-soins/ou-et-comment/les-crcm-et-carte-des-centres-de-soins). France also initiated a census of CF patients in 1990 which evolved into a modern registry in 2007. National registry reports are routinely published and shared publically, including reports of center level process and outcome measures (http://www.vaincrelamuco.org/face-la-mucoviscidose/registre-et-muco-en-chiffres/valorisation-des-donnees). There are currently 7000 people with CF in France, over half of whom are adults (http://www.vaincrelamuco.org/2014/12/21/registry-and-cystic-fibrosis-figures-294).

In 2004, the French Ministry of Health launched the first national plan for rare diseases aimed at not only invigorating research on rare diseases, but also recognizing the national CF CERD to lead cross-cutting activities. In 2006 CF diagnostic and treatment standards were issued and two CF CERDs, one at the Hospices Civils de Lyon and one bi-site at Nantes and Roscoff were certified. The Nantes-Roscoff CF CERD action plan featured the following priorities: information and communication system, therapeutic patient education, clinical research (in humanities and social sciences and in transplantation), and a program for care quality improvement. This plan was adopted by all CF center leaders in 2007 with a commitment to pursue center-level improvement in medical outcomes within 5 years. Between 2008 and 2011, leaders from the Nantes-Roscoff CF CERD and the French CF Association approached the US CFF and TDI to serve as mentors to enable the French CF community to adapt the U.S. initiative. This report outlines the specific lessons from the U.S. experience applied in the French CF care network.

## Methods

The Nantes-Roscoff CF CERD physician leader was first introduced to the improvement activities taking place in the U.S. by attending sessions at the annual North American CF Conference (NACFC). In addition to his lead role at the Nantes-Roscoff CF CERD, he served as principle in the development of the French CF Registry and director of the CF center in Roscoff and the western France CF care network. Between 2003 and 2007 he attended plenary sessions delivered by US CFF leaders, improvement experts, and parents. These sessions provided visibility to the US CFF’s initiative and emerging results: *"Accelerating the Improvement of CF Clinical Care"* presented Bruce Marshall, MD and Gerald O’Connor, PhD, ScD (2003); *"Care Providers and People with CF: Together We Can Make Great Things Happen!"* presented by Paul Batalden, MD, Jim Acton, MD, and Honor Page (2004), and *"Improving Patient Outcomes Using the Tools We Have Now"* presented by Michael Boyle, MD (2007) (www.cff.org). Symposia, workshops, and poster sessions at the NACFC showcased data transparency and public reporting and center-level improvement activities [8–19].

At the European CF Society Conference in 2007, the Nantes-Roscoff CF CERD leader and US CFF Senior Vice-President of Medical Affairs met in-person to discuss establishing a series of regular conference calls to explore the adaption of the improvement efforts taking place in the U.S. in France. The Nantes-Roscoff CF CERD leader had organized funding for a 6-month sabbatical to the U.S. and a visit to France by the US CFF Senior Vice-President to present at a national French CF Association conference in 2008. The US CFF Senior Vice-President suggested CF programs highly engaged in improvement activities to visit and facilitated introductions with these sites in preparation for the sabbatical. The US CFF Senior Vice-President also introduced the French leader to the TDI team (CF Quality Improvement Program Manager and The Dartmouth Institute Microsystem Academy (TDIMA) Co-Founder) and invited them to join the regular meetings and to share CF-specific quality improvement course material and invitations to participate as an observer in the U.S. CF improvement sessions being led by TDI on behalf of the US CFF (http://clinicalmicrosystem.org/about/our-partners/).

In addition to conducting site visits during his 2008 sabbatical, the Nantes-Roscoff CF CERD leader participated in the 10-week TDI Clinical Microsystems course and met bi-weekly with the TDI team to learn more about their role in supporting national efforts, specifically organizing the Learning and Leadership Collaborative and CF Quality Coaching Program [20]. In 2010, CF Quality Improvement Program Manager was invited to present at a French CF Association planning meeting. She and the Nantes-Roscoff CF CERD leader presented an overview of Clinical Microsystems (http://clinicalmicrosystem.org) and its adaption by the US CFF.

In 2011, the French CF Association supported a health care professional (physiotherapist) and a parent, affiliated with the association board and a quality engineer by training, to return to the U.S. for 2 months along with the Nantes-Roscoff CF CERD leader. This team conducted benchmarking site visits and enrolled in the 10-week TDI Clinical Microsystems course and the TDIMA Coaching Program [21]. The CF Quality Improvement Program Manager was invited to present an update on US CFF activities at the 2011 national CF conference and to meet one-on-one with center-level improvement teams. Regular meetings and visits between the US CFF, TDI, and French team continue. A timeline is presented in Table 1.

**Table 1:** US CF Foundation, The Dartmouth Institute, and French CF Leadership Partnership Timeline

Date	Event	Purpose
October 2006	NACFC	French CF leaders attend QI sessions.
June 2007	European CF Society Conference	US CFF and French CF leaders meet to organize a sabbatical for the Nantes-Roscoff CF CERD leader.
February–June 2008	Nantes-Roscoff CF CERD Leader U.S. Sabbatical	Participate in strategic meetings at the US CFF; site visit 5 US CF centers; attend QI training at Dartmouth and US CFF QI learning collaborative.
March 2008	National French CF Meeting	US CFF leader invited to present *Accelerating the Rate of Improvement in* CF *Care Initiative*.
September 2010	National French CF Meeting	Dartmouth leader presents US CFF Initiative progress; agrees to collaborate to launch PHARE-M.
October 2010	NACFC	Dartmouth and French leaders agree to support a French QI team in formal QI training at Dartmouth, adaptation of material, and participation in a US CFF QI learning collaborative.
March 2011	National French CF Meeting	Dartmouth leader presents Initiative progress; site visits 2 French CF centers; reviews PHARE-M progress.
April–September 2011	French QI team U.S. Sabbatical	Site visit 4 US CF Centers; attend QI training at Dartmouth and US CFF QI learning collaborative; complete adaptation of US QI material.
September 2011–June 2012	PHARE-M Pilot	7 French CF Centers participate; Dartmouth leader attends the collaborative kick-off.
September 2012–June 2013	PHARE-M 2	8 French CF centers participate.
May 2013	National Canadian CF Meeting	Dartmouth and French QI leaders meet to share progress; French team site visits 3 Canadian CF Centers.
September 2013–December 2014	PHARE-M Standardization	French QI curriculum receives national recognition as a professional development program.
January–December 2015	PHARE-M 3	4 French CF centers participate.
July 2014– June 2015	PHARE-M Research Project	14 French CF centers participate in an evaluation and audit.

While there is no formal program, the US CFF does periodically meet with and attend conferences organized by international CF organizations to share research as well as clinical care standards and improvement. The UK CF Trust, CF Canada, and CF Australia have also reached out to the US CFF to learn about quality improvement efforts. Financial support for the activities described in the manuscript was provided the French CF Association. The US CFF provides an annual grant to TDI to support its role in the national quality improvement initiative, and both CFF and TDI leaders were given support to collaborate with the French team.

## Results

A community of practice was cultivated through the regular exchange of ideas, implementation decisions, and activities between the US CFF, TDI, and the French team. Adapted to their local context, the French team organized a leadership structure for improvement that included representation for all stakeholders in the CF community and the government to advise and sanction the initiative. The team also actively sought to learn first-hand from improvement experts, and front-line teams, to ensure fidelity to methodology and translation between the U.S. and French CF care context. US CFF and TDI colleagues were invited to communicate about improvement activities and lessons in-person with French CF leaders and CF teams to support and endorse their efforts.

### Leadership for improvement

In 2007, after regular meetings between the Nantes-Roscoff CF CERD team and leaders in the French CF Association and the French CF Society, a call to action to provide exemplary care for all individuals with CF was adopted by all French CF centers. The national CERD formed a steering committee of clinicians, researchers, and parents from each organization and included a physician lead of the national French Patient Therapeutic Education program to gain government approval, to launch PHARE-M program. This committee commissioned registry analyses and drafted a national charter to steer reporting from the registry that mandated linkages between patient outcomes and quality improvement goals to inform and activate the CF community. They hired the parent with a background in quality engineering and who participated in the quality improvement and coaching training as an improvement coordinator to oversee and manage execution of the initiative. This committee continues to direct PHARE-M program and members meet regularly with the US CFF and TDI to seek advice and to learn of new activities being undertaken in the U.S. such as the deployment of a national Patient and Family Experience of Care Survey and development of a dashboard to enable the coproduction of CF care [22–24].

### Direct observation of frontline improvement teams

The Nantes-Roscoff CF CERD leader visited 6 centers in 2008 (Denver, Salt Lake City, Seattle, Chicago, Akron and Madison) and with 2 members of the standing committee (physiotherapist and parent) visited 4 centers in 2011 (Burlington, Akron, Minneapolis and Chicago). Two site visits were convened in conjunction with a Learning and Leadership Collaborative session facilitated by TDIMA. These opportunities enabled the French team to participate in the didactic learning sessions of the collaborative and the special sessions convened for CF Quality Coaches.

Through interviews with CF center clinicians and direct observation these visits gave the team an appreciation for the role of local leaders in creating the conditions for personnel to acquire the knowledge and skills for improvement; using data to inform improvement; and, the role of external coaching to facilitate learning and achieving center goals. The team collected examples of system assessments (staff and patient satisfaction surveys, task and time trackers, and measures of staffing patterns), data collection tools and run-charts, as well as examples of treatment algorithms and patient education materials that helped teams standardize patient care, e.g. nutrition and pulmonary care pathways, chronic medication checklists, disease screening tools.

The French team developed a draft quality improvement curriculum, including content and material, connecting the Clinical Microsystems model for improvement and its application in a CF care center because of these visits. The drafts were shared with the PHARE-M program steering committee to further adapted to the French CF care context and system.

### Immersion in Clinical Microsystems

As students, the French team studied the theory of Clinical Microsystems and participated in the practicum of working with a clinical team to improve care. This experience exposed the team to the teaching and didactic methods that they used to translate and adapt CF specific improvement examples for teams in France. The materials included slide decks and workbooks covering assessing practice, setting aims, running tests of change, and measurement. The team also took advantage of participating in the TDIMA Coaching Program to learn how to create action plans and timelines and offer encouragement to frontline teams.

This deep immersion into the theory and practice of improvement not only facilitated the adaption of material, such as the CF Action Guide and the US CFF curriculum [3] to the French CF care center context, but also expedited the development of the team’s knowledge and skills to lead and teach improvement in the French CF community. The French leadership team continues to participate in the TDI learning community, most recently participating in a workshop on Standards for Quality Improvement Reporting Excellence (SQUIRE2.0).

### Communicating new ideas and adapting the U.S. initiative

The committee convened to spearhead PHARE-M program invited US CFF and TDI leaders to France on 4 separate occasions to communicate and spread highlights and lessons from the U.S. initiative. The US CFF Senior Vice-President was invited to present at the national French CF Annual Conference in Marne-la-Vallée (March 2008). He communicated lessons from the U.S. initiative and met one-on-one with key opinion leaders at the French CF Association and their board. In March 2012, the Co-Founder of TDIMA was invited to serve as an expert advisor to the PHARE-M program at the face-to-face meeting in Marseille to provide guidance and expertise on the poster presentation of the 7 center teams involved in the first collaborative. The French team continues to rely the Co-Founder’s expert guidance.

The CF Quality Improvement Program Manager was invited by the PHARE-M committee to participate in a 3-day planning retreat at the headquarters of the French CF Association in 2010 in Paris. This meeting was convened to review center data, define roles, and draft a work plan for the coming year. In 2011, the program manager returned to France to meet one-on-one with teams at two CF centers forming improvement teams to participate in the PHARE-M program, to speak at the French CF Association Annual Conference in Reims and meet with individuals with CF and families.

Following the sabbaticals and visits from US CFF and TDI leaders, the French team completed the initial adaption of the U.S. initiative. They articulated a vision for improving care; adapted patient centered goals supported by data from their patient registry; published an improvement guide; and engaged CF center teams, including individuals with CF and families, in a learning quality improvement collaborative.

## Discussion

### Success factors

The Nantes-Roscoff CF CERD, specifically the PHARE-M program committee, adapted the five critical success factors of the U.S. initiative to launch a national program: issuing a strategic plan with a call to action, committing as an organization to a culture of improvement, investing in the capacity of professionals to engage in improvement, partnering with individuals with CF and families, and integrating improvement into the system of CF care [2]. Within the context of the French health care system, French leaders successfully navigated and partnered with governing bodies to enact appropriate policies and secure resources to embark on improving care for individuals with CF. They prioritized hiring and investing to develop staff to serve as national leaders and coordinators to execute the improvement initiative and regularly convened with U.S. leaders to seek input and advice. They engaged care center teams, individuals with CF and their families in their efforts to improve care, tackling the continuum of CF care including transition from pediatrics to adult care and lung transplantation (cf. articles A08, A09 in the OJRD Supplement) and enhancing patient education activities. They continue to spread these improvement activities across their network of care centers.

### Future considerations

While the French leaders did adapt the critical success factors of the U.S. initiative there remain elements that could be deployed to continue to enrich and accelerate improvement efforts. Pursuing a plan to standardize registry data capture, and provide timely reporting, will facilitate care management, inform center-level improvement activities, and allow transparency and benchmarking opportunities both in France and other European countries with similar health care systems [25]. The French CF leaders could consider establishing discipline-specific mentoring programs to engage professionals new to CF care in both learning more about CF and promoting quality improvement [7]. It may also be worth exploring deployment of a national survey to capture first-hand the patient and family care experience to supplement process and outcomes registry data and to more deeply engage individuals with CF and families in improvement [22].

### Implications

The French team demonstrated an unwavering interest in adapting the US CFF improvement strategies to improve the quality and length of life for people with CF in France. They approached the US CFF eager to learn and committed time and resources. The US CFF and TDI generously shared their experience and expertise and dedicated their time to help the French team. This international collaboration demonstrates the advantages of building a community of practice to engage in collective learning and regular interaction. Such communities can result in transformative change and improvement.

## Conclusions

The Nantes-Roscoff CF CERD team successfully adapted the US CFF’s initiative to accelerate improvement in CF care by establishing a partnership with U.S. leaders to communicate and exchange strategies and lessons learned; intentionally studying and adapting the Clinical Microsystems approach to quality improvement; and learning directly from the experience of frontline teams in the U.S. They continue to partner with U.S. leaders and are seeking to collaborate with European colleagues to continue to improve care for individuals with CF and their families across Europe.
